# Identification and Characterization of a Novel d‐Branched‐Chain Amino Acids Importer from *Lactobacillus fermentum*

**DOI:** 10.1002/cbic.202401075

**Published:** 2025-02-27

**Authors:** Koichiro Aoki, Yuta Mutaguchi, Hisashi Hemmi, Tohru Yoshimura, Tomokazu Ito

**Affiliations:** ^1^ Department of Applied Biosciences Graduate School of Bioagricultural Sciences Nagoya University Furou-chou, Chikusa, Nagoya, Aichi 464–8601 Japan; ^2^ Department of Biotechnology Faculty of Bioresource Sciences Akita Prefectural University Akita Japan

**Keywords:** d-amino acid, d-branched-chain amino acids, Lactic acid bacteria, Transporter

## Abstract

Various lactic acid bacteria synthesize d‐branched‐chain amino acids (d‐BCAA) during growth, but their physiological function remains largely elusive. The pyridoxal phosphate‐dependent enzyme isoleucine 2‐epimerase (ILEP) has been identified as the key enzyme responsible for d‐BCAA biosynthesis. Comparative genomic analyses revealed that genes encoding ILEP and an uncharacterized amino acid‐polyamine‐organocation (APC) family transporter are adjacent in several d‐BCAA‐producing bacteria, suggesting a functional link between these two proteins in d‐BCAA metabolism. In this study, we investigated the function of the APC family transporter from *Lactobacillus fermentum* (*Lf*AAP). Using heterologous expression systems in *Escherichia coli* and *Lactococcus lactis*, we demonstrated that *Lf*AAP functions as a non‐stereospecific BCAA importer. Mutational analysis revealed that Ala119 and Met331 play critical roles in substrate recognition. Heterologous expression of *Lf*AAP and/or *Lf*ILEP in a *L. lactis* strain, which lacks the ILEP‐AAP genes operon, revealed that ILEP functions as both synthetic and catabolic enzyme for d‐BCAA. Our findings suggest that the ILEP‐AAP system contribute to storage and subsequent utilization of BCAA in a form that is less accessible by other organisms, providing a potential competitive advantage in microbial environments.

## Graphical abstract

Certain lactic acid bacteria produce d‐branched‐chain amino acids (d‐BCAA) and encode isoleucine 2‐epimerase (ILEP) and an uncharacterized transporter (AAP). We demonstrate that the transporter is a novel, non‐stereospecific BCAA importer. The ILEP‐AAP operon likely aids in storing and utilizing BCAA in a form less accessible to other organisms.

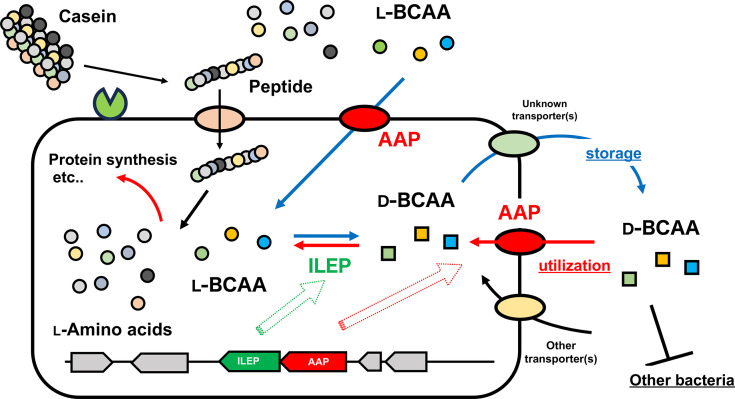

## Introduction

In bacteria, d‐amino acids serve critical roles, primarily as components of cell wall peptidoglycan and certain antibiotics. The most common d‐amino acids found in bacterial peptidoglycan are d‐Ala and d‐Glu, which are located at the 5th and 7th positions respectively in the peptidoglycan stem peptide.[^1^, ^2^] These d‐amino acids play important role in enhancing the physical and biological strength of the peptidoglycan. Some other d‐amino acids, such as d‐Asp, d‐Ser or d‐Lys, have been found in the peptidoglycans of certain bacteria, including *Lactococcus lactis*,^[3]^ *Enterococcus faecium*,^[4]^ *Enterococcus gallinarum*,^[5]^ vancomycin‐resistant *Staphylococcus aureus*,^[6]^ and *Thermotoga maritime*.^[7]^ Recent studies revealed that a wide variety of bacteria produce diverse d‐amino acids and utilizes them in various biological processes beyond peptidoglycan biosynthesis, such as cell wall remodeling and maintenance, environmental stress response, biofilm control, and/or defense mechanism.[^2^, ^8^, ^9^, ^10^, ^11^, ^12^] These functions enable bacteria to adapt and survive under diverse environmental conditions.

Previous studies revealed that certain lactic acid bacteria synthesize and accumulate d‐branched‐chain amino acids (d‐BCAA), including d‐*allo*‐Ile, d‐Leu, and d‐Val, in the cultivation medium. During the growth of such lactic acid bacteria, the d‐BCAA concentrations in the medium reaches sub‐millimolar concentration.[^13^, ^14^] The physiological significances of d‐BCAA, however, remain largely elusive. A pyridoxal phosphate‐dependent enzyme, isoleucine 2‐epimerase (ILEP), was identified in *Lactobacillus buchneri* (Uniprot ID: M1GRN3), which catalyzes interconversion of l‐BCAA and their corresponding d‐BCAA.^[15]^ Recent study has shown that several lactic acid bacteria, such as *Limosilactobacillus fermentum*, *Limosilactobacillus vaginalis*, *Limosilactobacillus reuteri*, and *Weissella paramesenteroides*, encode the ILEP homolog that shares more than 50 % sequence identity with *L. buchneri* ILEP. These species have also been found to produce d‐BCAA, suggesting that the key role of ILEP homologs in d‐BCAA biosynthesis.^[14]^

In *L. buchneri*, ILEP is encoded by the *Lbuc_2316* gene, which is adjacent to the *Lbuc_2315* gene encoding a putative amino acid–polyamine–organocation (APC) family transporter.^[16]^ This genomic arrangement suggest that two genes are likely forming an operon. Interestingly, comparative genomic analyses showed that several d‐BCAA‐producing *Lactobacillus* species also exhibit a similar genomic structure, raising the possibility that the transporter plays a role in d‐BCAA production.

In this study, we characterized the APC family transporter from *L. fermentum* (*Lf*AAP) using a heterologous expression system with *Escherichia coli* and *Lactobacillus lactis*. We first demonstrated that the transporter functions as a proton‐coupled, non‐stereospecific importer of BCAA. Our data show that, together with ILEP, the transporter plays a role in facilitating the storage and subsequent utilization of BCAA in a form that is not readily available to competing organisms, thereby providing a competitive advantage in nutrient‐limited environment.

## Results

### Comparative Genomic Analysis Reveals the Functional Link Between ILEP and an APC Family Transporter

**Figure 1:**
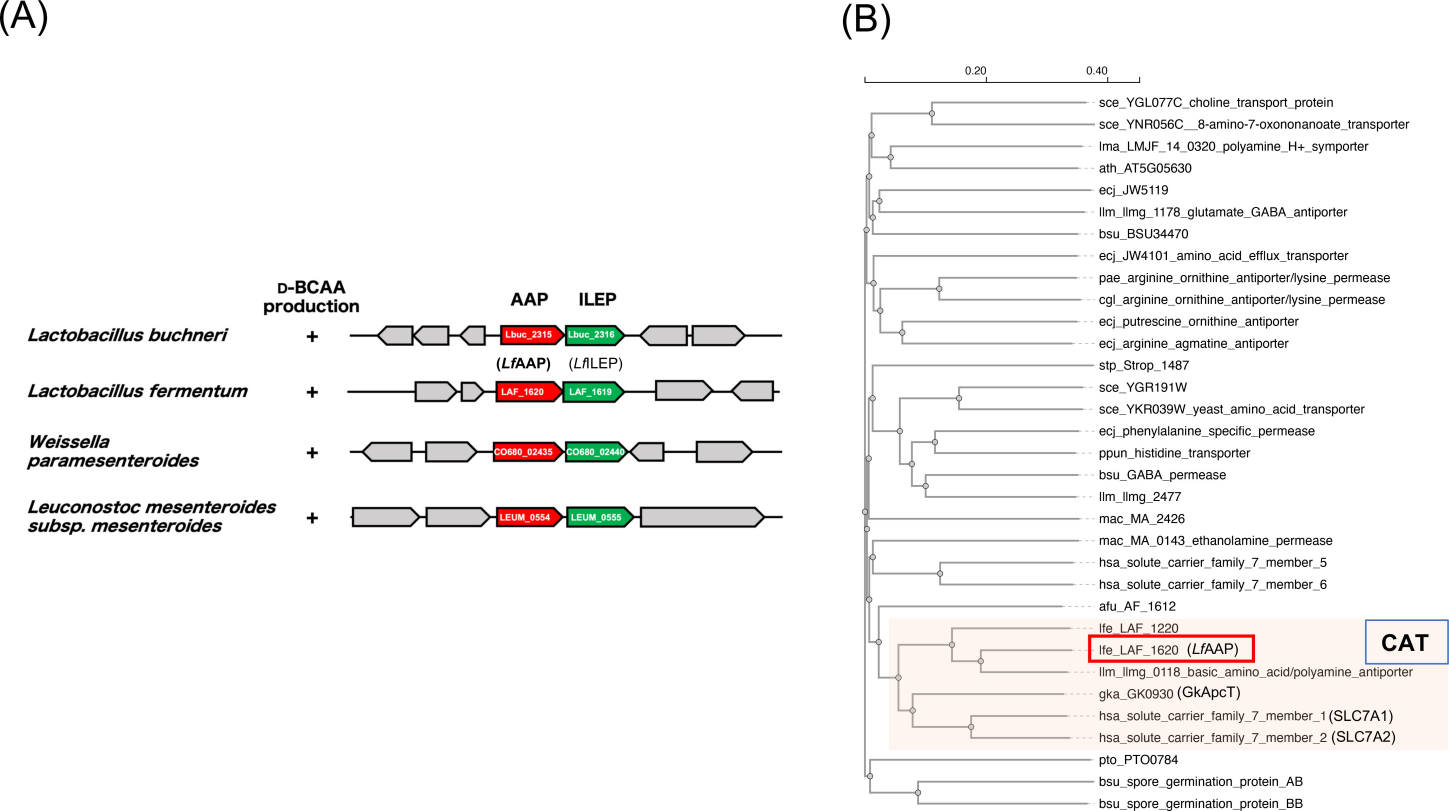
Distribution and characterization of *Lf*AAP and its homologs. (A) Genomic structure around ILEP gene in lactic acid bacteria. The genomic arrangement of the ILEP gene (green) and its flanking genes is depicted. In many d‐BCAA‐producing lactic acid bacteria, an APC family transporter gene (red) is located adjacent to the ILEP gene, suggesting a potential functional relationship. (B) Phylogenic analysis of *Lf*AAP. The amino acid sequences of *Lf*AAP (lfe_LAF_1620) and 30 characterized APC family transporters were aligned by CLUSTALW (https://www.genome.jp/tools‐bin/clustalw) and then phylogenic tree was constructed with Molecular Evolutionary Genetics Analysis (MEGA) software.^[37]^ *Lf*AAP is within the cluster of the cationic amino acid transporter (CAT) subfamily clade of the APC family transporters. Representative members of the CAT subfamily include SLC7A1 (solute carrier family 7 member 1; UniProt: P30825), SLC7A2 (solute carrier family 7 member 2; UniProt: P52569), and GkApcT from *G. kaustophilus* HTA426 (GK0930; UniProt: Q5L1G5).

In *L. buchneri*, ILEP is encoded by the *Lbuc_2316* gene, which is adjacent to the *Lbuc_2315* gene that encodes a putative APC family transporter, and these two genes probably form an operon (Figure 1A). Proteins belonging to the APC family are found in various organisms, ranging from bacteria to humans, and function as symporters, uniporters, or antiporters for a wide range of metabolites.[^16^, ^17^, ^18^] Interestingly, several *Lactobacillus* species that produce d‐BCAA, such as *Lactobacillus mesenteroides*, *Lactobacillus fermentum*, and *Weissella paramesenteroides*, exhibit a similar genomic arrangement: a gene encoding an ILEP homolog and an adjacent gene encoding a putative transporter of the APC family (Figure 1A). These observations suggest that the ILEP homologs and adjacent transporters may play a crucial role in d‐BCAA production in these lactic acid bacteria.

Based on these genomic insights, we investigated the function of the transporter and selected LAF_1620 from *L. fermentum* NBRC 3956, whose complete genome is available, as a model protein. This transporter, named *Lf*AAP, consists of 484 amino acids with 12 predicted transmembrane segments. In the *L. fermentum* genome, the *Lf*AAP gene is situated adjacent to LAF_1619 gene (*Lf*ILEP), which encodes an ILEP that shares 56 % sequence identity with the *L. buchneri* ILEP, and the two genes likely form an operon.

To explore the evolutionary relationships of *Lf*AAP within the APC family of transporters, we conducted a phylogenetic analysis using 30 characterized APC family transporters representing 15 subfamilies. *Lf*AAP was clustered within the phylogenetic clade of the cationic amino acid transporter (CAT) subfamily of the APC family (Figure 1B). Representative members of this subfamily include human SLC7A1 (solute carrier family 7 member 1; UniProt: P30825), SLC7A2 (solute carrier family 7 member 2; UniProt: P52569), and a bacterial homolog from *Geobacillus kaustophilus* HTA426 (GK0930; UniProt: Q5L1G5). These transporters are known to mediate the uptake of small, hydrophobic, and/or polar amino acids.[^19^, ^20^] These findings support the hypothesis that *Lf*AAP functions as a transporter for d‐BCAA.

### *Lf*AAP does not Function as d‐BCAA Exporter

Currently, little information is available regarding d‐BCAA exporters. One plausible function of LfAAP is to act as an exporter of d‐BCAA. To test this hypothesis, we used a heterologous expression system in *E. coli* cells, which lack ILEP and do not produce d‐BCAA. If *Lf*AAP functions as a d‐BCAA exporter, co‐expression of *Lf*ILEP and *Lf*AAP would enable *E. coli* cells to synthesize and potentially excrete higher levels of d‐BCAA.

We constructed three *E. coli* strains with arabinose‐inducible expression system for *Lf*AAP and/or *Lf*ILEP: one overexpressing *Lf*ILEP (designated BW/ILEP2^+^ *)*, another expressing the *Lf*AAP (BW/AAP2^+^), and a third co‐expressing both *Lf*ILEP and *Lf*AAP (BW/AAP‐ILEP^+^). A strain carrying empty vectors (BW/ev2) served as a control. These strains were cultured in M9‐glucose medium containing l‐BCAA, and the d‐BCAA levels in the medium were analyzed.

**Figure 2:**
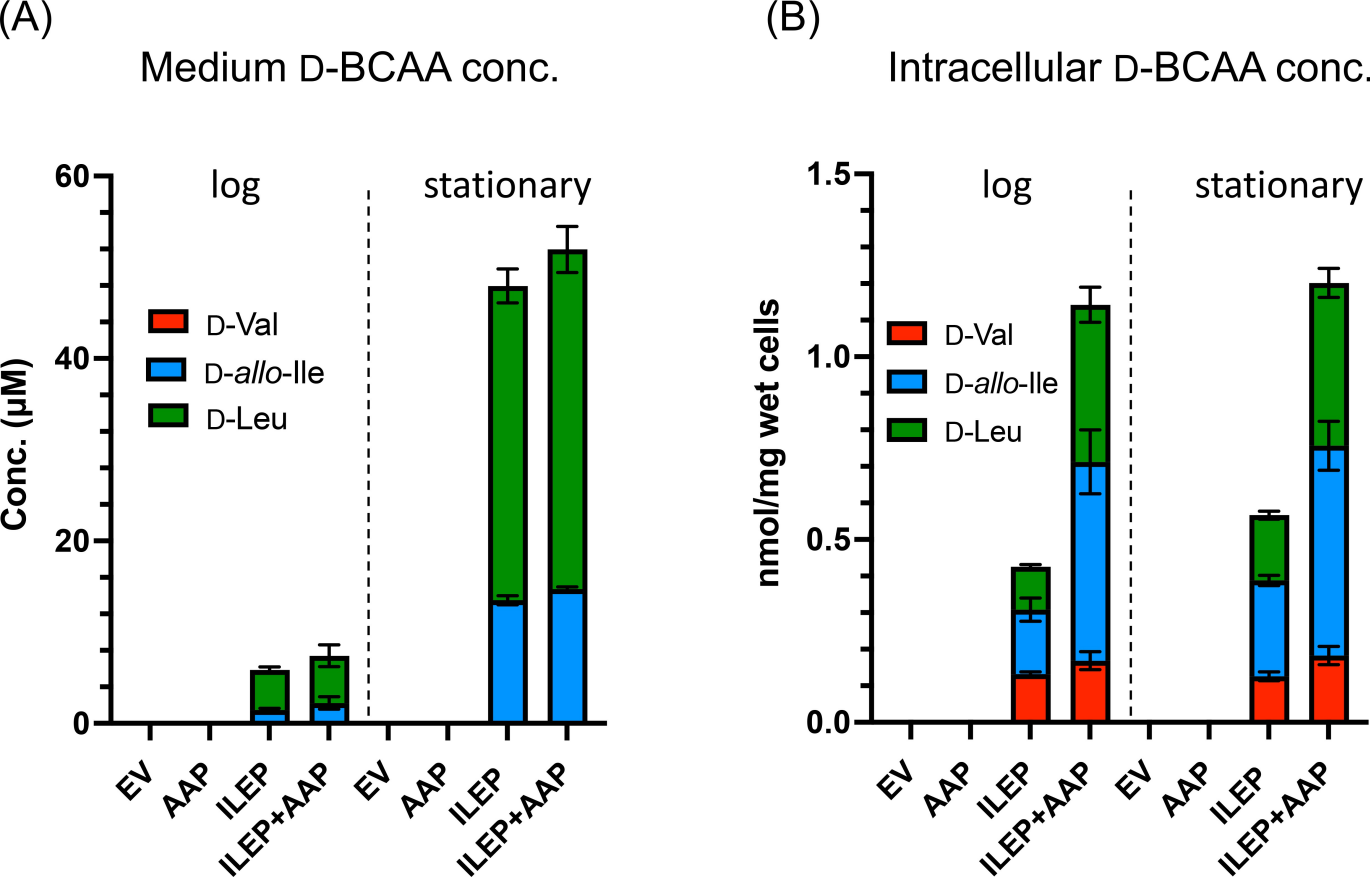
Effect of *Lf*AAP and/or *Lf*ILEP expression in *E. coli* on extracellular and intracellular d‐BCAA levels. Wild‐type strains carrying either empty vectors (BW/ev2), *Lf*ILEP‐expression vector (BW/ILEP2^+^), *Lf*AAP‐expression vector (BW/AAP2^+^), or *Lf*AAP and *Lf*ILEP‐expression vectors (BW/AAP‐ILEP^+^) were grown in M9 medium supplemented with antibiotics and 0.02 % l‐arabinose at 37 °C. Samples of culture media and cell pellets were collected during the log phase (OD_600_=0.6) and stationary phase (OD_600_=1.0). The levels of d‐BCAA in the media (A) and cells (B) were quantified by HPLC, as detailed in the Materials and Methods section. All experiments were performed in triplicate and error bars indicate standard deviation.

As shown in Figure 2A, no d‐BCAA was detected in the media of BW/ev2 and BW/AAP2^+^. In contrast, d‐Leu and d‐*allo*‐Ile were detected in the media of BW/ILEP2^+^ and BW/AAP‐ILEP^+^, with nearly identical d‐BCAA concentrations for both strains. This observation aligns with previous findings demonstrating the efficient conversion of l–Leu and l–Ile to d‐Leu and d‐*allo*‐Ile by *Lf*ILEP.^[14]^ While these results confirm the functionality of *Lf*ILEP as a d‐BCAA biosynthetic enzyme within the *E. coli* system, they do not provide evidence to support a role for *Lf*AAP in facilitating d‐BCAA export under the experimental conditions tested.

**Figure 3:**
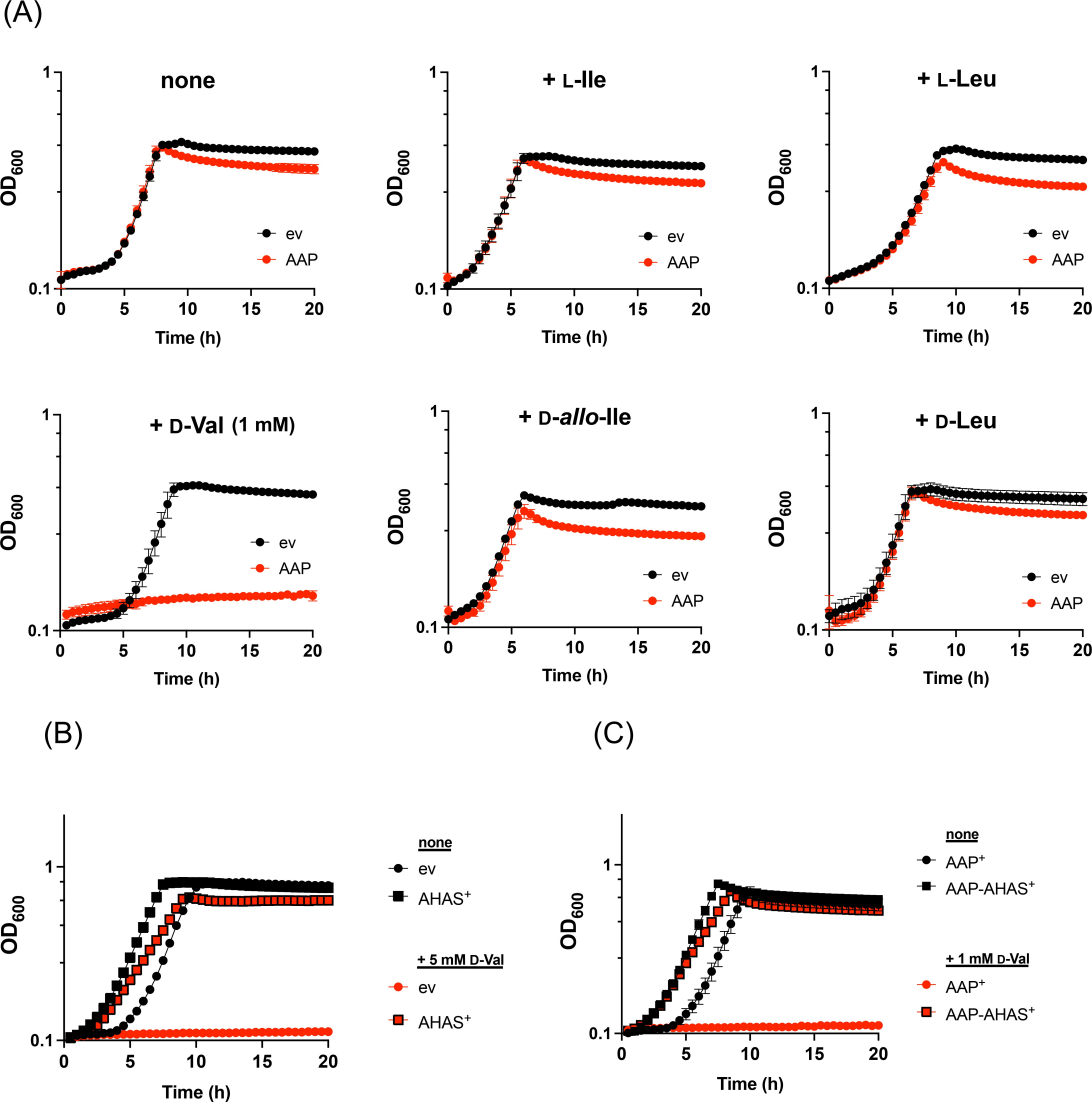
Effect of *Lf*AAP expression on the *E. coli* growth in the presence or absence of BCAA. (A) Wild‐type *E. coli* strains carrying an empty vector (BW/ev) or overexpressing *Lf*AAP (BW/AAP^+^) were cultured in M9‐glucose medium supplemented with chloramphenicol, 0.02 % l‐arabinose, and 5 mM of either l–Ile, l–Leu, or d‐*allo*‐Ile, or 1 mM of d‐Val. l–Val exhibited significant toxicity to both BW/ev and BW/AAP^+^ strains, completely inhibiting growth at concentrations exceeding 10 μM (data not shown). (B) BW/ev3 and BW/AHAS^+^ strains were cultured in M9‐glucose medium supplemented with antibiotics and 0.02 % l‐arabinose, in the presence or absence of 5 mM d‐Val. (C) BW/AAP3^+^ and BW/AAP‐AHAS^+^ strains were similarly cultured in M9‐glucose medium supplemented with antibiotics and 0.02 % l‐arabinose, with or without 1 mM d‐Val. All cultures were incubated at 37 °C with continuous shaking for 20 hours, and OD_600_ values were recorded every 30 min using the Epoch2 microplate spectrophotometer. Error bars represent standard deviations from triplicate experiments.

We conducted growth assays using *E. coli* strains carrying an empty vector (BW/ev) or expressing *Lf*AAP (BW/AAP^+^) in the presence or absence of various amino acids. In M9 synthetic medium, both strains exhibited similar growth rates, however, the stationary‐phase optical density (OD) was slightly lower for BW/AAP^+^ strain (Figure 3A). Supplementation with l–Ile, l–Leu, d‐*allo*‐Ile, or d‐Leu to the medium had no significant impact on the growth rate of either strain. In contrast, significant growth differences were observed in the presence of d‐Val. While BW/ev grew robustly in medium containing 1 mM d‐Val, the BW/AAP^+^ strain failed to grow under these conditions. These findings suggest that *Lf*AAP expression enhances d‐Val toxicity in *E. coli*, likely by facilitating its uptake rather than its export.

The *E. coli* K‐12 strain is highly sensitive to l–Val due to its inhibitory effect on acetolactate synthases (AHAS I and AHAS III), enzymes essential for l‐BCAA biosynthesis.[^21^, ^22^] Based on this, we hypothesized that d‐Val might exert similar inhibitory effects on AHAS enzymes. Indeed, the addition of 5 mM exogenous d‐Val completely inhibited *E. coli* growth, regardless of *Lf*AAP expression (Figures 3B and C). Moreover, the expression of l–Val‐insensitive acetolactate synthase II (AHAS II) conferred resistance to d‐Val toxicity, abolishing its inhibitory effect in both wild‐type and *Lf*AAP‐overexpressing *E. coli* strains (Figures 3B and C). These findings support the hypothesis that *Lf*AAP mediates d‐Val uptake, leading to AHAS inhibition and subsequent growth impairment in *E. coli*.

### *Lf*AAP Function as a Proton‐Coupled d‐BCAA Importer

We analyzed intracellular amino acid pools of BW/ev2, BW/ILEP2^+^, BW/AAP2^+^, and BW/AAP‐ILEP^+^ strains cultured in M9‐glucose medium containing l‐BCAA. As expected, no d‐BCAA was detected in the BW/ev2 or BW/AAP2^+^ cells. In contrast, both *Lf*ILEP‐expressing strains, BW/ILEP2^+^ and BW/AAP‐ILEP^+^, accumulated d‐BCAA (d‐Val, d‐*allo*‐Ile, and d‐Leu) in the cells, confirming that *Lf*ILEP functions as a d‐BCAA‐biosynthetic enzyme. Importantly, the intracellular d‐BCAA levels in the WT/AAP‐ILEP^+^ were approximately twice as high as those in the BW/ILEP2^+^ without affecting the levels of other amino acids (Figure 2B). These findings suggest that *Lf*AAP facilitates uptake of d‐BCAA into the cells.

**Figure 4:**
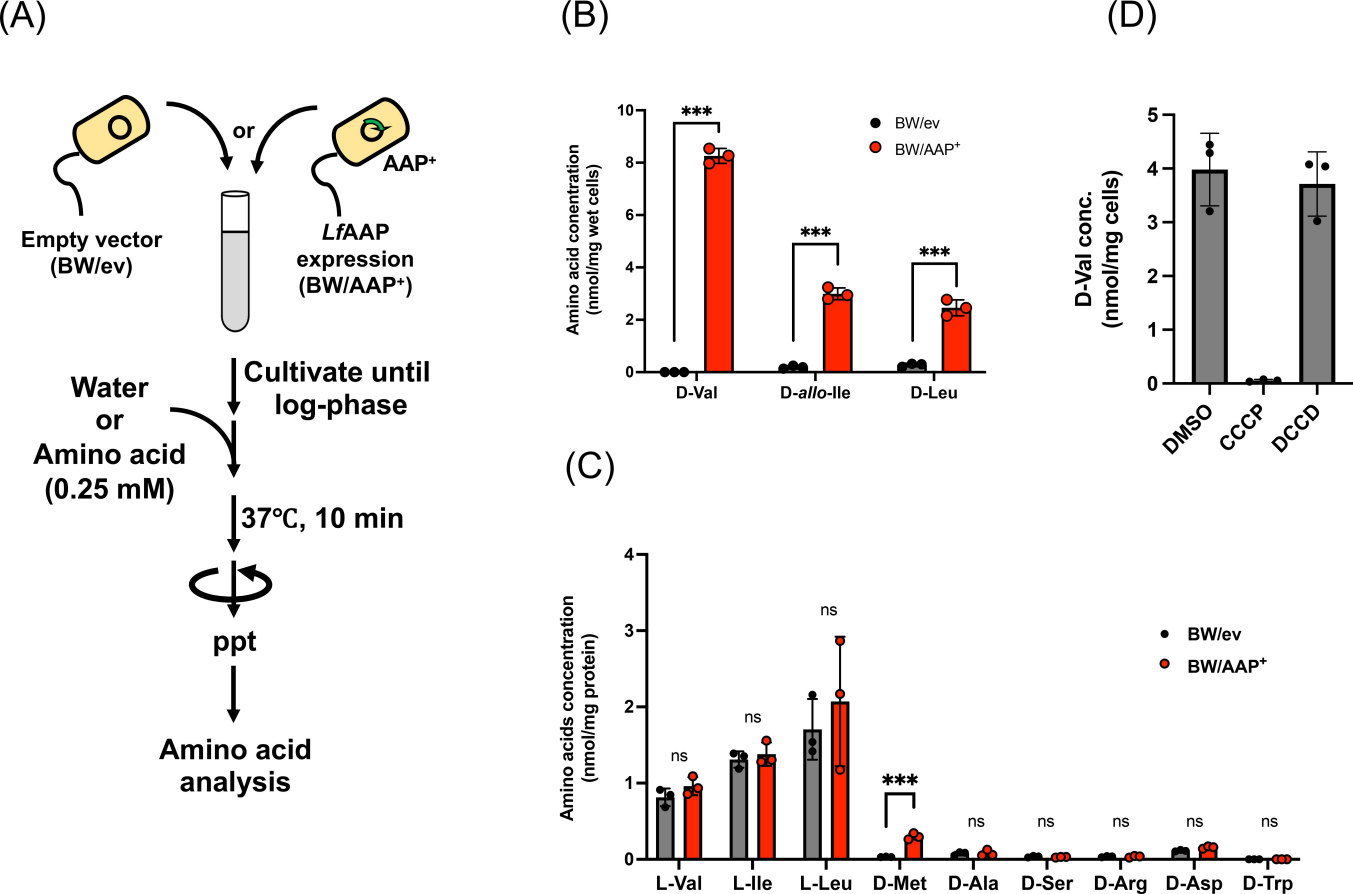
Characterization of *Lf*AAP‐mediated d‐BCAA uptake. (A) Schematic depiction of the procedure of the amino acid uptake assay. BW/ev and BW/AAP^+^ were grown in M9‐glucose medium supplemented with Cm, 0.02 % l‐arabinose at 37 °C with shaking to the log phase. Amino acid was added at a final concentration of 0.25 mM. After 10 minutes of incubation, the increase in intracellular amino acid concentration was determined by HPLC. (B) Effect of *Lf*AAP expression on the uptake of d‐BCAA. Increase of intracellular d‐Val, d‐*allo*‐Ile, or d‐Leu upon supplementation of d‐Val, d‐*allo*‐Ile, or d‐Leu. (C) Substrate specificity of *Lf*AAP. Uptake of various amino acids, including d‐Ala, d‐Arg, d‐Asp, d‐Met, d‐Ser, d‐Trp, l–Val, l–Ile, and l–Leu, was assessed. (D) Effect of CCCP or DCCD on *Lf*AAP‐mediated d‐Val uptake. d‐Val and DMSO (vehicle), CCCP, or DCCD were added to the culture medium of BW/AAP^+^ at the log phase, and the increase in intracellular d‐Val levels was determined. All experiments were conducted in triplicate. Asterisks indicates statistical significance determined using Student's t‐test (***P<0.001). The ns indicates no significant difference.

To directly evaluate the d‐BCAA uptake capacity of *Lf*AAP, we performed amino acid uptake experiments. Strains expressing an empty vector (BW/ev) or *Lf*AAP (BW/AAP^+^) were grown to the log‐phase in M9‐glucose medium, supplemented d‐BCAA (d‐Leu, d‐Val, or d‐*allo*‐Ile), and intracellular d‐BCAA concentrations were determined (Figure 4A). When d‐Val was supplemented, the BW/ev cells had negligible d‐Val, while BW/AAP^+^ cells accumulated significantly higher d‐Val (8.26±0.23 nmol/mg wet cells). Similarly, supplementation of d‐*allo*‐Ile or d‐Leu led to dramatic increase in the intracellular d‐BCAA concentration only in the BW/AAP^+^ strain, with approximately 16‐fold and 9‐fold higher levels, respectively, compared to BW/ev. These results demonstrate that *Lf*AAP functions as a d‐BCAA importer (Figure 4B).

The substrate specificity of *Lf*AAP was further assessed using various d‐amino acids, including d‐Ala, d‐Arg, d‐Asp, d‐Met, d‐Ser, or d‐Trp. No increase in the intracellular concentration of d‐Ala, d‐Arg, d‐Asp, d‐Ser, or d‐Trp was observed both in BW/ev and WT/AAP^+^. However, supplementation with d‐Met resulted in BW/AAP^+^ accumulating ~9.4 times more d‐Met than BW/ev, indicating that *Lf*AAP can also transport d‐Met (Figure 4C).

To evaluate if *Lf*AAP can transport l‐BCAA, we conducted amino acid transport assays with l‐BCAA. Unlike the case with d‐amino acids, exogenous l‐BCAA significantly increased the intracellular concentration of l‐BCAA regardless of the presence or absence of *Lf*AAP, probably due to endogenous l‐BCAA transport activity.[^23^, ^24^] No difference in the intracellular l‐BCAA concentration was observed between BW/ev and BW/AAP^+^ under the condition examined (Figure 4C), supporting the conclusion that the *Lf*AAP lacks l‐BCAA transport activity. However, we observed decreased d‐Val uptake in the presence of l–Ile. *E. coli* possesses endogenous l‐BCAA transport system (both for uptake and efflux) and metabolic enzymes,[^23^, ^25^] which may obscure the measurement of l‐BCAA transport activity by *Lf*AAP in our *E. coli* system. These situations preventing a definitive conclusion on whether *Lf*AAP can transport l‐BCAA.

Various *Lf*AAP homologs employ different mechanisms for amino acids transport. In mammals, CAT family transporters function as exchangers or facilitators, while SCL7 family transporters in plants operates in a pH‐dependent manner.^[26]^ A recent study identified an amino acid transporter from *G. kaustophilus* (GkApcT), which shares 36 % sequence identity with *Lf*AAP, as a proton‐coupled transporter for small hydrophobic and polar amino acids, including l–Val, l‐Ala, l‐Thr, l‐Ser, l‐Asn, l‐Tyr, and d‐Ala (listed in order of uptake efficiency).^[20]^ To assess whether *Lf*AAP functions as a proton‐coupled transporter, we performed d‐Val uptake assays in the presence or absence of the proton ionophore carbonyl cyanide *m*‐chlorophenyl hydrazone (CCCP)^[27]^ or the ATP synthase inhibitor *N,N*‐dicyclohexylcarbodiimide (DCCD).^[28]^ *Lf*AAP‐mediated d‐Val transport was completely abolished by CCCP but remained unaffected by DCCD (Figure 4D). These findings indicate that *Lf*AAP functions as a proton‐coupled d‐BCAA importer.

### Characterization of the *Lf*AAP Transport Mechanism

**Figure 5:**
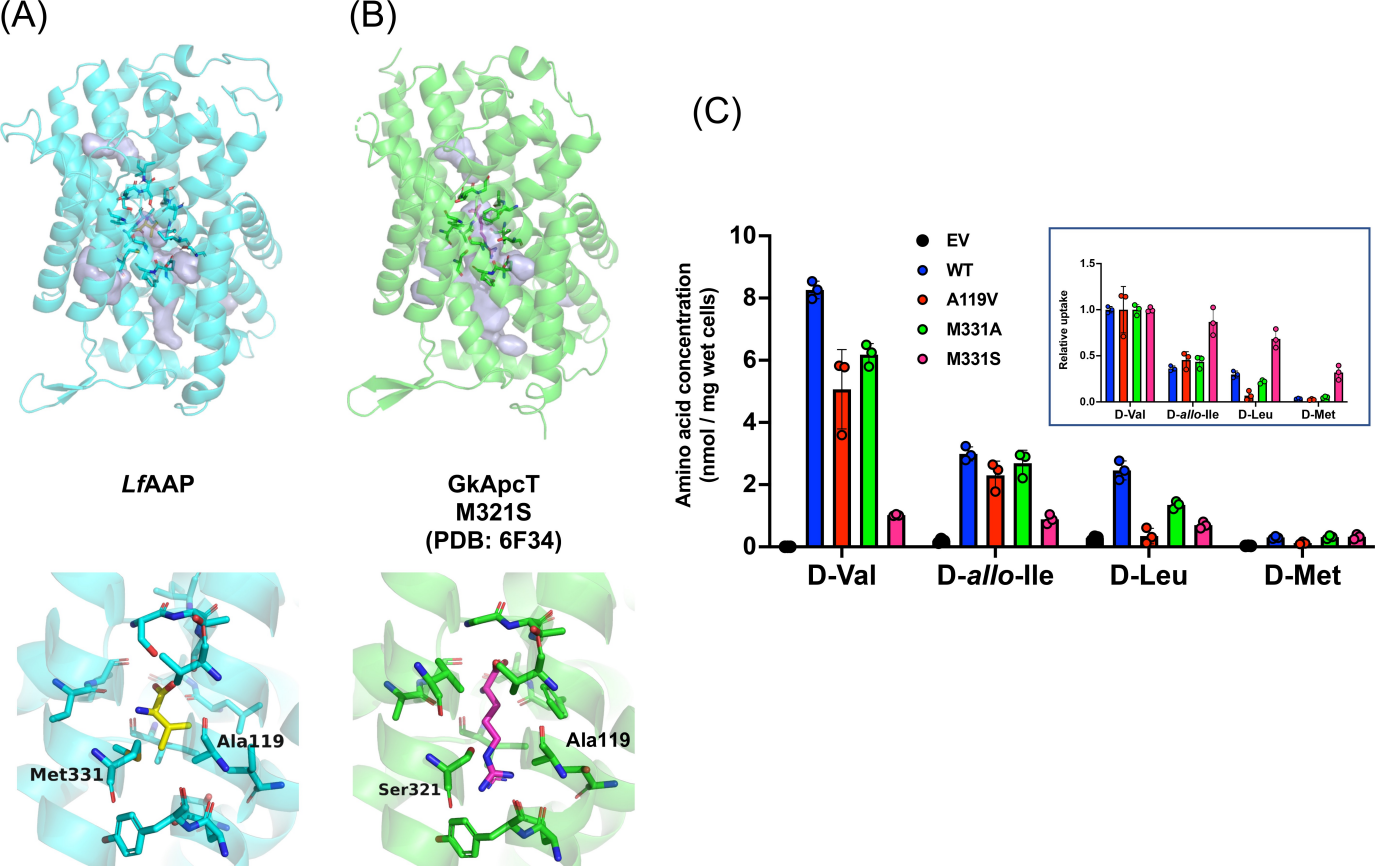
Molecular basis of BCAA specificity of *Lf*AAP. (A) AlphaFold2 model of *Lf*AAP and its putative d‐Val binding site. The putative binding mode of d‐Val was predicted using AutoDock Vina,^[38]^ with the binding mode of l‐Ala in GpApcT (PDB ID 5OQT) as a reference. Ala119 and Met331 are positioned near the side chain binding region for d‐Val, suggesting a role in recognition of side chain of d‐BCAA. (B) The overall structure and substrate binding site of the M331S mutant of GpApcT bound to l‐Arg (PDB ID: 6F34). The M321S mutation in GkApcT is known to confer the ability to transport basic amino acids such as l‐Arg, as shown.^[20]^ (C) The effect of A119V, M331A, or M331S mutation on *Lf*AAP‐mediated d‐Val uptake. Strains expressing wild‐type or mutated *Lf*AAP (BW/AAP^+^, BW/AAP^A119V+^, BW/AAP^M331A+^, or BW/AAP^M331S+^) were grown to the log‐phase, followed by supplementation with 0.25 mM of either d‐Val, d‐*allo*‐Ile, d‐Leu or d‐Met. The increase in intracellular d‐amino acid levels was determined by HPLC. The inset figure depicts the relative uptake of each d‐amino acid with respect to d‐Val uptake in each strain. All experiments were conducted in triplicate. Error bars represent standard deviations.

*Lf*AAP is the first example of a d‐BCAA importer. To obtain insight into the molecular basis of its substrate specificity, the putative amino acid binding site of *Lf*AAP was predicted using docking simulations. An AlphaFold2 model of *Lf*AAP was employed with the crystal structure of the *Gk*ApcT complexed with l‐Ala serving as a reference structure. This analysis suggests that the protein backbone, along with the side chains of Ser42, Val123, Ala119, Leu240, and Met331, is involved in substrate recognition. Notably, Ala119 and Met331, positioned near the side chain binding region for d‐Val, likely contribute to the recognition of the side chains of d‐BCAA in *Lf*AAP (Figure 5A). Supporting this, a previous study showed that mutating M321 in *Gk*ApcT (the equivalent of M331 in *Lf*AAP) to serine enabled the transporter to recognize and transport basic amino acids, such as l‐Arg and l–Lys (Figure 5B).^[20]^

To identify amino acid residues involved in substrate recognition by *Lf*AAP, we generated A119V, M331A, or M331S mutants and conducted amino acid uptake assay with d‐Val, d‐*allo‐*Ile, d‐Leu, and d‐Met. The wild‐type *Lf*AAP exhibits a substrate preference order of d‐Val>d‐*allo*‐Ile>d‐Leu>d‐Met, based on the increase in the intracellular d‐amino acid concentration. The A119V mutant retained the ability to transport d‐Val and d‐*allo*‐Ile, but specifically lost the ability to transport bulky amino acids, such as d‐Leu and d‐Met (Figure 5C). The M331S mutant exhibited a broader substrate specificity for d‐BCAA and demonstrated more efficient transport of d‐amino acids with longer side chains, such as d‐Met and d‐Leu, compared to the wild‐type protein (Figure 5C, inset). Note that the M331S mutant did not facilitate the transport of positively charged amino acids, such as d‐Arg and d‐Lys (data not shown). The M331A mutant displayed a substrate specificity profile that was nearly identical to that of the wild‐type protein. These findings demonstrate that Ala119 and Met331 in *Lf*AAP are critical for fine‐tuning its substrate specificity.

### *Lf*AAP is a Non‐Stereospecific BCAA Importer

The lactic acid bacterium *Lactococcus lactis* lacks ILEP‐AAP operon and does not produce d‐BCAA during growth. To further investigate the function of the *Lf*AAP, an *L. lactis* NZ9000 strain expressing *Lf*AAP under a nicin‐inducible promoter (Ll/AAP^+^) and a control strain carrying an empty vector (Ll/ev) were constructed. Amino acid uptake assays were then performed using various d‐ and l‐amino acids to evaluate *Lf*AAP‐mediated amino acid transport.

**Figure 6:**
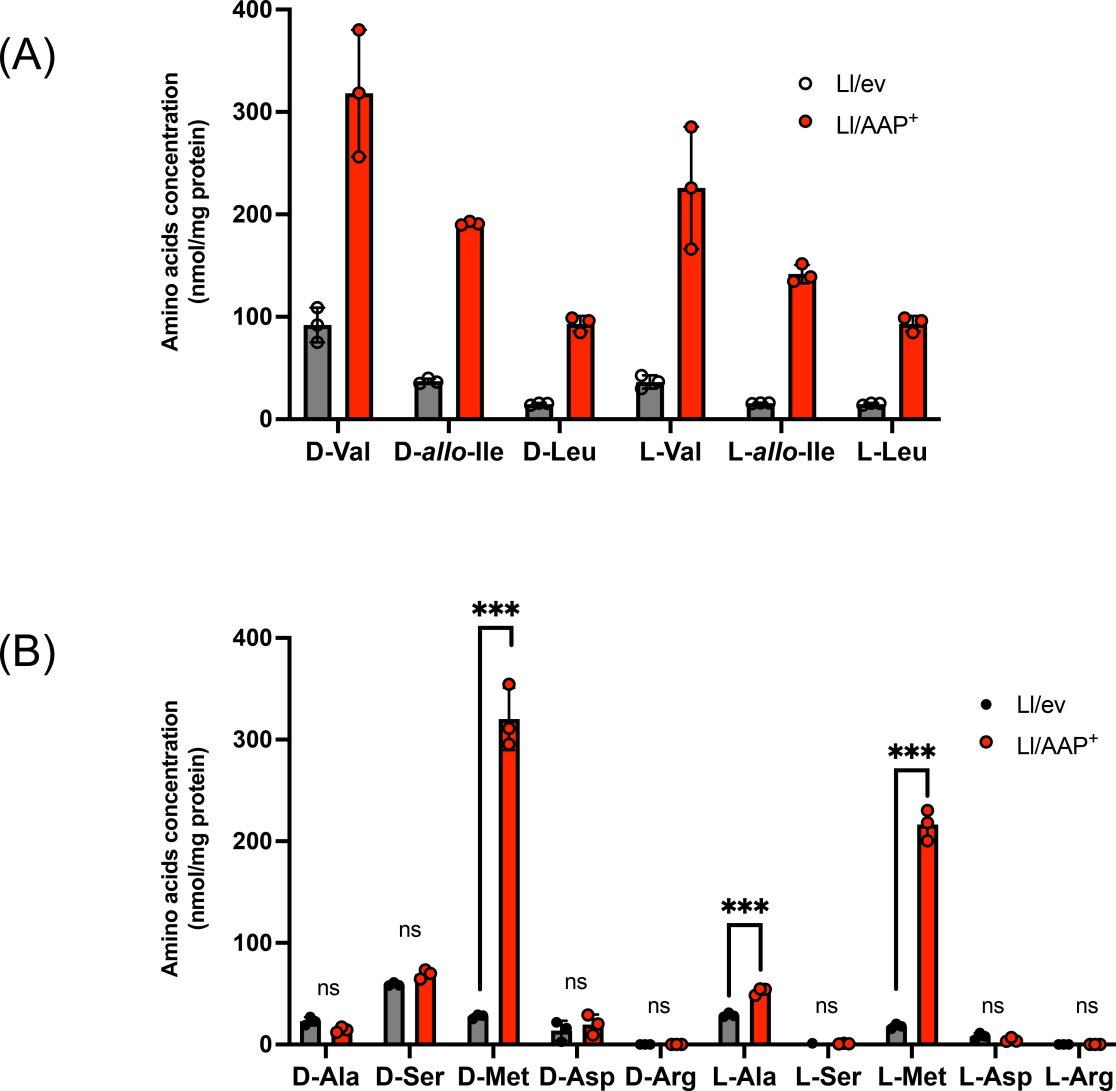
Effect of *Lf*AAP expression on amino acid uptake in *L. lactis. L. lactis* strain expressing *Lf*AAP (Ll/AAP^+^) or the control strain (Ll/ev) were grown to the log‐phase. After centrifugation and medium exchange, a 1 mL bacterial suspension was prepared. Each suspension was supplemented with 0.5 mM of the respective amino acid and incubated for 10 minutes. Intracellular amino acid concentrations were measured using UHPLC and normalized to the protein concentration in the cell extracts (nmol/mg protein). Amino acid used were (A) d‐, or l–Val, (*allo*)‐Ile, Leu, and (B) d‐, or l–Met, Ala, Ser, Asp, or Arg. Detailed procedures are provided in the Materials and Methods section. Asterisks indicates statistical significance determined using Student's t‐test (***P<0.001). The ns indicates no significant difference.

As shown in Figure 6A, supplementation with d‐BCAA (d‐Val, d‐*allo*‐Ile, or d‐Leu) significantly increased intracellular d‐BCAA in both *L. lactis* strains, with Ll/AAP^+^ accumulating 3~5‐fold higher levels compared to Ll/ev. This result confirmed that *Lf*AAP functions as a d‐BCAA importer in *L. lactis* cells. Additional experiments with other d‐amino acids revealed that supplementation with d‐Met leads to significantly higher intracellular d‐Met levels in Ll/AAP^+^ compared to Ll/ev. However, the uptake of d‐Ala, d‐Ser, d‐Asp, and d‐Arg was comparable between the two strains (Figure 6B). These results demonstrated that *Lf*AAP specifically facilitates the import of d‐BCAA and d‐Met in *L. lactis* cells.

Similar experiments using l‐amino acids revealed that, upon supplementation with l‐BCAA (l–Val, l–Ile, or l–Leu) or l–Met, Ll/AAP^+^ accumulated significantly higher levels of these amino acids compared to Ll/ev, demonstrating that *Lf*AAP facilitates the transport of l‐BCAA and l–Met. Notably, the extent of intracellular l‐BCAA accumulation was comparable to that observed with d‐BCAA (Figure 6A). These findings confirm that *Lf*AAP functions as a non‐stereospecific BCAA importer, capable of transporting both d‐ and l‐BCAA enantiomers with similar efficiency.

### ILEP‐AAP Operon is Responsible for d‐BCAA Production, Storage, and Utilization

Our findings suggest that the ILEP‐AAP operon plays a dual role in d‐BCAA metabolism, supporting both production and utilization. AAP functions as a non‐stereospecific BCAA importer, while ILEP catalyzes the racemization of BCAA, converting l‐BCAA into d‐BCAA and *vice versa*. This system is proposed to (i) produce d‐BCAA through the import and racemization of l‐BCAA, followed by the export of d‐BCAA into the medium via unidentified mechanism(s), and (ii) utilize d‐BCAA by importing it and converting it back into l‐BCAA.

**Figure 7:**
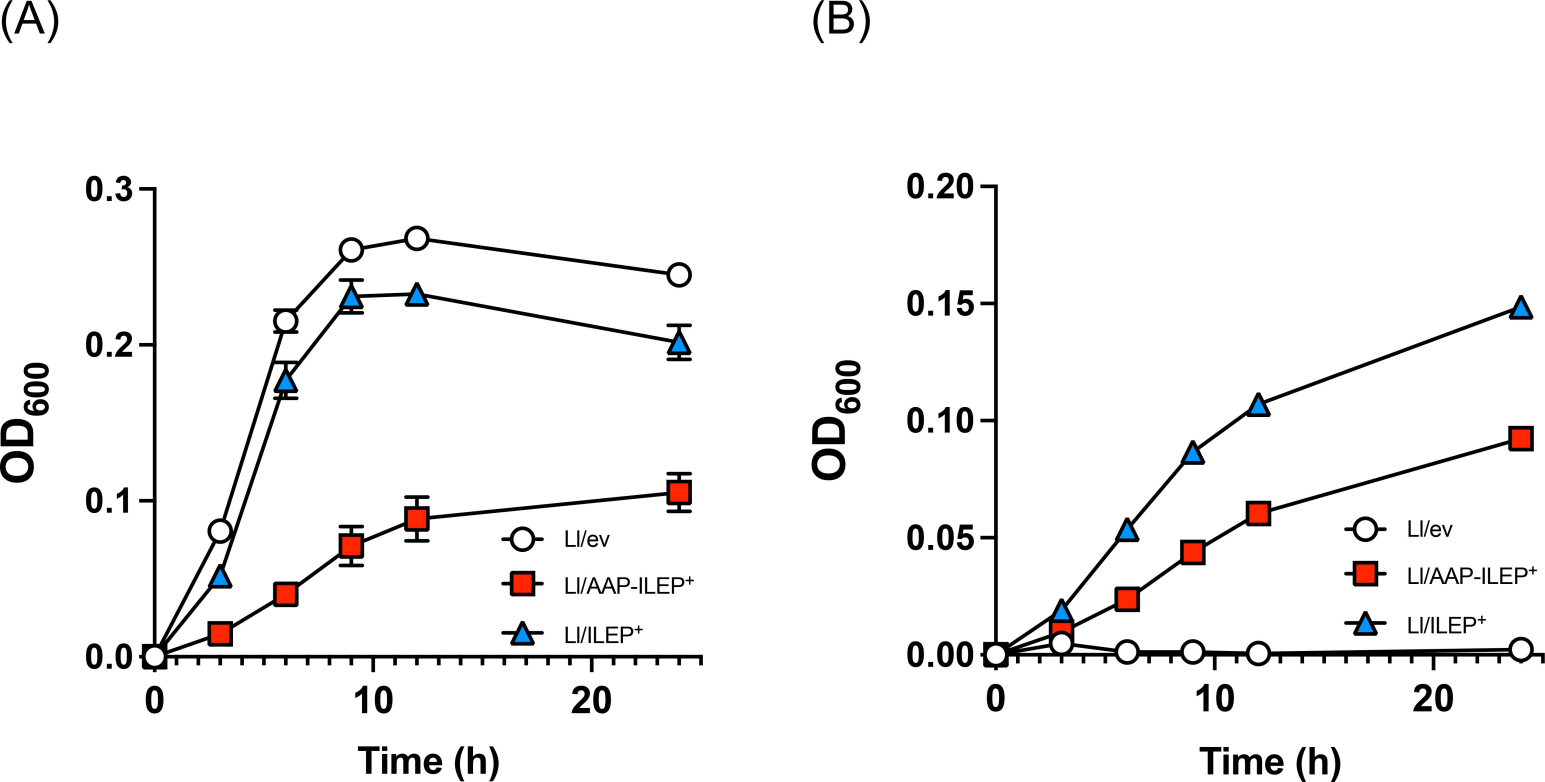
Role of AAP‐ILEP operon on BCAA utilization in lactic acid bacteria. *L. lactis* strain expressing *Lf*ILEP (Ll/ILEP2^+^) and *Lf*ILEP*‐Lf*AAP (Ll/AAP‐ILEP^+^) or the control strain (Ll/ev) were grown in a semi‐defined medium containing l‐BCAA (A) or d‐BCAA (B). The cultures were incubated at 30 °C, and growth was monitored at OD_660_ using a microplate reader. Error bars represent standard deviations from triplicate experiments.

To test the role of this operon in d‐BCAA utilization, we expressed *Lf*ILEP and/or *Lf*AAP in *L. lactis* NZ9000, a strain lacking both the ILEP‐AAP operon and the *de novo* pathway for l‐BCAA synthesis. Growth assays in synthetic media revealed that all *L. lactis* strains grew robustly in the presence of l‐BCAA, regardless of *Lf*ILEP or *Lf*AAP expression (Figure 7A). However, in synthetic media containing d‐BCAA as the sole BCAA source, only strains expressing *Lf*ILEP could grow (Figure 7B). This finding demonstrates that ILEP expression enables *L. lactis* to utilize d‐BCAA by converting it into l‐BCAA.

In contrast, *Lf*AAP did not show a clear positive contribution to BCAA catabolism under the conditions examined (Figure 7A and B). As shown in Figure 6A, the *L. lactis* strain has an intrinsic ability to take up both l‐BCAA and d‐BCAA, which may have masked the specific contribution of *Lf*AAP in our experimental setup. Rather, the *L. lactis* strain co‐expressing *Lf*AAP and *Lf*ILEP exhibited a slower growth rate than the strain expressing *Lf*ILEP alone. The underlying mechanism remains unclear; however, *Lf*AAP expression resulted in an ~10‐fold increase in intracellular levels of both enantiomers of BCAA and Met (Figure 6), which may have negatively impacted *L. lactis* growth.

## Conclusions

Using heterologous expression systems in *E. coli* and *L. lactis*, we identified the first d‐BCAA importer from *L. fermentum*, *Lf*AAP. Our findings demonstrated that *Lf*AAP functions as a proton‐coupled, non‐stereospecific importer of BCAA, where Ala119 and Met331 playing critical role in determining substrate specificity.

**Figure 8:**
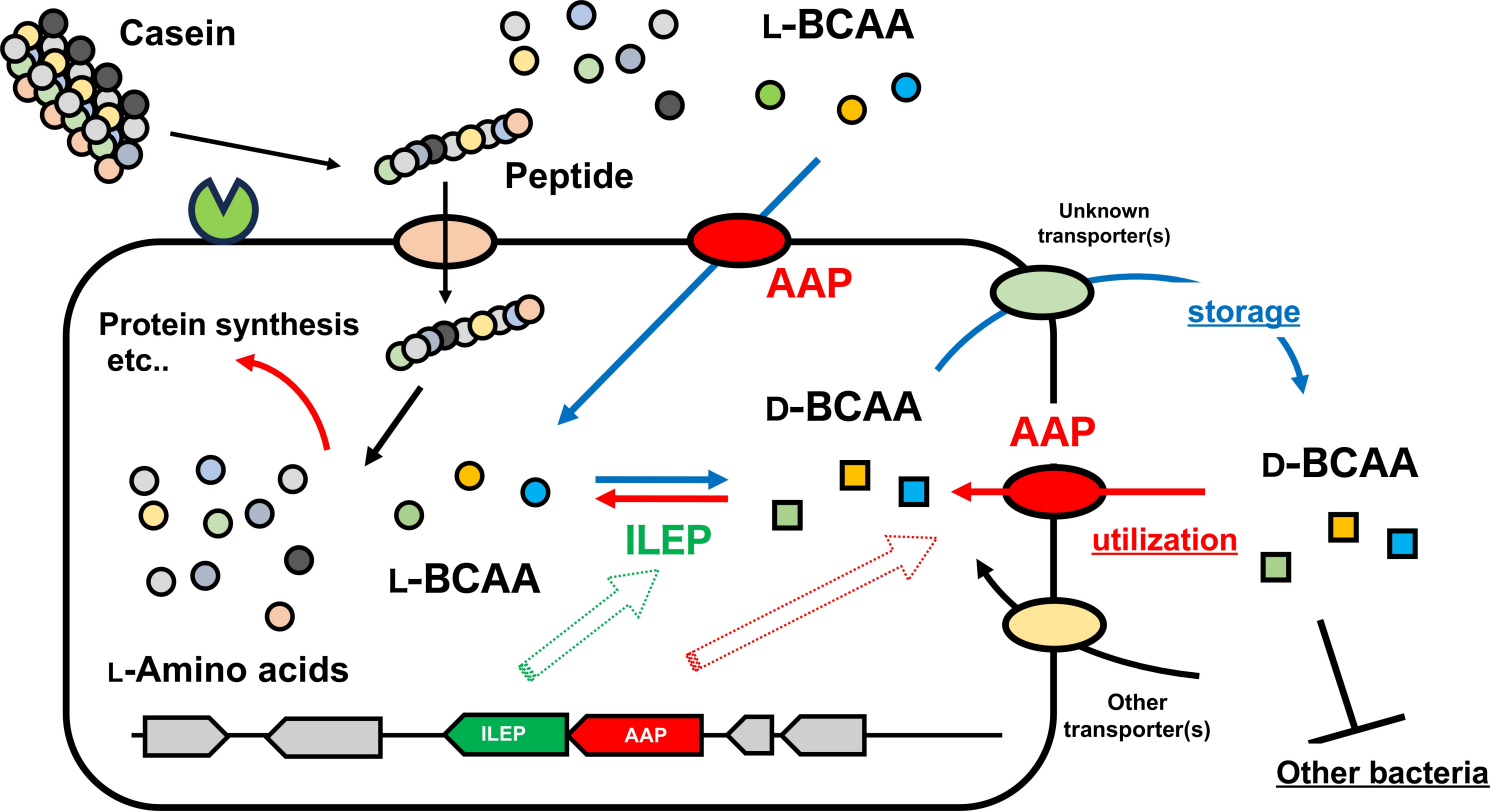
Proposed role of AAP‐ILEP operon in lactic acid bacteria. The AAP‐ILEP operon plays a dual function in the synthesis and utilization of d‐BCAA. Initially, l‐BCAA are imported via AAP (and/or potentially other mechanism), converted into d‐BCAA by ILEP. d‐BCAA are subsequently exported into the medium (indicated by blue arrows). Since d‐BCAA is less accessible to competing organisms, it acts as a storage form of BCAA. When metabolic demands arise, the same system facilitates the reuptake of d‐BCAA, with ILEP converting it back into l‐BCAA for cellular utilization (red arrows). This process ensures a highly adaptable and efficient use of BCAA. By enabling effective storage and reutilization of BCAA, the AAP‐ILEP operon provides lactic acid bacteria with a significant competitive advantage in diverse and challenging ecological niches.

Based on these finding, we propose that the AAP‐ILEP operon serves a dual function in the synthesis and utilization of d‐BCAA (Figure 8). Specifically, l‐BCAA are initially imported via AAP and converted into d‐BCAA by ILEP. d‐BCAA acts as a storage form of BCAA that is less accessible to competing organisms. Since only limited bacteria, including *L. fermentum*, can utilize d‐BCAA for the growth–due to the restricted distribution of d‐BCAA catabolic enzymes such as ILEP, d‐amino acid dehydrogenase, and d‐amino acid transaminase – this system can provide a competitive edge. Subsequently, when required, the same proteins facilitate the reuptake of d‐BCAA and their conversion back into l‐BCAA, enabling an adaptable and efficient use of BCAA for metabolic needs. Notably, *Lf*ILEP exhibits significantly lower catalytic efficiency towards Val,^[14]^ while *Lf*AAP efficiently transports Val. This suggest that the AAP‐ILEP system operates cooperatively to preferentially elevate intracellular Val concentrations and facilitate the efficient interconversion of Val enantiomers, comparable to other BCAA.

This mechanism is particularly advantageous for lactic acid bacteria that lack a *de novo* pathway for l‐BCAA biosynthesis. For example, *L. fermentum* is auxotrophic for l–Val, l–Leu, and l–Ile, due to the absence of ketol‐acid reductoisomerase, an enzyme required for the reduction of 2‐acetolactate and 2‐aceto‐2‐hydroxybutyrate to their corresponding dihydroxy acids, intermediates in BCAA biosynthesis. For these organisms, acquiring BCAA from their environment is a critical survival strategy.

The AAP‐ILEP system thus provides a competitive advantage in nutrient‐limited and/or closed environments by enabling efficient storage and utilization of BCAA. These findings will highlight the unique metabolic adaptations of lactic acid bacteria and provide insights into the ecological strategies employed by lactic acid bacteria in competitive microbial ecosystems.

## Materials and Methods

*limosilactobacillus fermentum* NBRC 3956 was provided by the RIKEN BRC through the National BioResource Project (MEXT, Japan). *lactococcus lactis* NZ9000 was purchased from MoBiTec GmbH (Germany). Amino acids were purchased from Wako‐Fujifilm or Kanto Chemical. Primers were from Hokkaido System Science.

### Construction of Plasmids

Plasmids used in this study are summarized in Table 1. Primers for plasmid construction are listed in Table 2. The *Lf*AAP (LAF_1620, NCBI‐ProteinID: BAG27956) expression vector, pAEA16, was constructed as follows. The *Lf*AAP gene was amplified with primers LAF_1620‐fw and LAF_1620‐rv using the genomic DNA of *L. fermentum* NBRC 3956 as template. The plasmid backbone was amplified by PCR with primers pBAD‐fusion‐up and pBAD‐fusion‐dwn using pAE1147 as template. The pAE1147 is derived from pBAD/myc‐HisA, in which the Amp^r^ gene and pBR322 origin were replaced with chloramphenicol resistant gene and p15A replication origin originated from pACYC177. These PCR products were purified from agarose gel, assembled as described previously,^[29]^ and introduced into *E. coli* Top10 cells. Similar procedures were used to construct the *Lf*ILEP (LAF_1619, NCBI‐ProteinID: BAG27955) expression vector (pAEA17) with the primer pairs LAF_1619‐fw and LAF_1619‐rv. The plasmid backbone was amplified with primers pBAD‐fusion‐up and pBAD‐fusion‐dwn using pBAD/myc‐HisA (Invitrogen) as template.

**Table 1:** Strains and plasmids used in this study.

Strain	Genotype, description	Reference or source
*E. coli* strains		
BW25113 (WT)	BW25113 [*rrnB* Δ*ara*BAD567 Δ*rha*BAD568 Δ*lac*Z4787 HsdR514 *rph*‐1]	Laboratory collection
BW/ev	BW25113 (WT) harboring pAE1147	This study
BW/ev2	WT harboring pAE1147 and pAE1080	This study
BW/ev3	WT harboring pAE1147 and pDM1599	This study
BW/AHAS^+^	WT harboring pAE1147 and pAEU91	This study
BW/AAP^+^	WT harboring pAEA16 (*Lf*AAP expression)	This study
BW/AAP2^+^	WT harboring pAEA16 and pAE1080	This study
BW/AAP3^+^	WT harboring pAEA16 and pAEU67	This study
BW/AAP‐AHAS^+^	WT harboring pAEA16 and pDM1599	This study
BW/ILEP^+^	WT harboring pAEA17 (*Lf*ILEP expression)	This study
BW/ILEP2^+^	WT harboring pAEA17 and pAE1080	This study
BW/AAP‐ILEP^+^	WT harboring pAEA16 and pAEA17 (*Lf*AAP and *Lf*ILEP expression)	This study
BW/AAP^A119V+^	WT harboring pAEA53	This study
BW/AAP^M331A+^	WT harboring pAEA56	This study
BW/AAP^M331S+^	WT harboring pAEA24	This study

*L. lactis* strains		
NZ9000	*L. lactis* NZ9000 (wild‐type)	MoBiTec GmbH
Ll/ev	NZ9000 harboring pNZ8148	Laboratory collection
Ll/AAP^+^	NZ9000 harboring p*Lf*AAP2	This study
Ll/ILEP^+^	NZ9000 harboring p*Lf*ILEP2	This study
Ll/AAP‐ILEP^+^	NZ9000 harboring p*Lf*AAP‐ILEP2	This study

Plasmids		
pAE1080	pBAD/Myc‐HisA	Invitrogen
pAE1147	pBAD/Myc‐HisA derivative, Cm^r^, p15A ori	Laboratory collection
pAEA16	pAE1080 encoding *Lf*AAP gene	This study
pAEA17	pAE1147 encoding *Lf*ILEP gene	This study
pAEA53	pAE1080 encoding *Lf*AAP A119V mutant	This study
pAEA56	pAE1080 encoding *Lf*AAP M331A mutant	This study
pAEA24	pAE1080 encoding *Lf*AAP M331S mutant	This study
pAEU67	pBAD24	Laboratory collection
pDM1599	pBAD24 encoding *ilvGM* (Val‐insensitive AHAS II)	[36]
pNZ8148	nicin inducible. *E. coli‐L.lactis* shuttle vector	This study
p*Lf*AAP2	pNZ8148 encoding *Lf*AAP gene	This study
p*Lf*ILEP2	pNZ8148 encoding *Lf*ILEP gene	This study
p*Lf*ILEP‐AAP2	pNZ8148 encoding *Lf*AAP‐*Lf*ILEP gene	This study

**Table 2:** Primers used in this study.

Name	Sequence (5′ to 3′)
for *E. coli* system	
LAF_1619*‐*fw	aacaggaggaattaaccatggaggagaaaaaaagtaaccagcag
LAF_1619*‐*rv	caatgatgatgatgatgatgttcccaacccagctcttctgcgttg
pBAd‐fusion‐up	catggttaattcctcctgttagccc
pBAd‐fusion‐dwn	catcatcatcatcatcattgagtttaaacgg
LAF_1620*‐*fw	aacaggaggaattaaccaatgagtttttggaaaaccattacgcgg
LAF_1620*‐*rv	caatgatgatgatgatgatgctttttttctcctccatgatcagtgtctc
LAF_1220*‐*fw	gctaacaggaggaattaaccatgaaacaattaatcgctcgt
LAF_1220*‐*rv	tcaatgatgatgatgatgatggcccttcttagccaaggt
LAF_1620^A119V^ *‐*up	gaaggccagcacgatgaagtactcggctaacagggcccag
LAF_1620^A119V^ *‐*dwn	tacttcatcgtgctggccttcgttgggtcggggctgtc
LAF_1620^M331A^ *‐*up	gccatgctcgccccgatcagggcggtgaacatccc
LAF 1620^M331A^ *‐*dwn	ctgatcggggcgagcatggccggctcccggctg
LAF_1620^M331S^ *‐*up	tgatcgggtctagcatggccggctcccggctg
LAF_1620^M331S^ *‐*up	gccatgctagacccgatcagggcggtgaac

for *L. lactis* system	
LfAAP_F2	agatcggaagagcacacgtctgaactccagtca
LfAAP_R2	agatcggaagagcacacgtctgaactccagtca
LfILEP_F2	aggcactcacatatggaggagaaaaaaagtaaccagcag
LfILEP_R2	agatcggaagagcacacgtctgaactccagtca
pNZ_F	gagctcaagctttctttgaaccaaaattag
pNZ_R	catatgtgagtgcctccttataatttattttgtag

The A119V, M331A, or M331S mutation was introduced by an overlapping PCR method with primer pairs LAF_1620^A119V^ *‐*up and LAF_1620^A119V^ *‐*dwn (for A119V mutation), LAF_1620^M331A^ *‐*up and LAF_1620^M331A^ *‐*dwn (for M331A mutation), LAF_1620^M331S^ *‐*up and LAF_1620^M331S^ *‐*dwn (for M331S mutation), respectively, with pAEA16 as template.

The expression vector of *Lf*AAP gene (p*Lf*AAP2), *Lf*ILEP gene (p*Lf*ILEP2), and both *Lf*AAP and *Lf*ILEP genes (p*Lf*AAP‐ILEP2) for *L. lactis* NZ9000 strain were constructed as follows. The full‐length *Lf*AAP gene, *Lf*ILEP gene, and the *Lf*AAP‐*Lf*ILEP gene cassette were amplified from the *l. fermentum* NBRC 3956 genome by PCR with the primers listed in Table 1. Additionally, the linear fragment of pNZ8148 was amplified by PCR using the primers pNZ_F and pNZ_R. The PCR‐generated inserts and the vector fragments were assembled by In‐Fusion Cloning Kit (Clontech) following the manufacturer's instructions.

In all cases, sequence of the insert gene was verified by sequencing.

### Bacterial Growth

Bacterial strains used in this study are listed in Table 1. The *E. coli* strains were precultured in LB medium supplemented with glucose (0.2 %), ampicillin (100 μg/ml), and/or chloramphenicol (30 μg/ml) at 37 °C overnight. The 500 μL of culture was collected by centrifugation (12,000 rpm, 1 min), washed twice with M9 medium, and resuspended in a 500 μL of M9‐glucose medium.^[30]^ For the growth assay, the 5 μL of bacterial suspension was inoculated to a 200 μL of the M9‐glucose medium containing appropriate antibiotics and various concentrations of amino acid. The bacterial strains were grown 37 °C with continuous shaking for 20 hours and Od_600_ values were recorded every 30 min using the Epoch2 microplate spectrophotometer (BioTek).

*L. lactis* NZ9000 strains harboring p*Lf*ILEP2, p*Lf*AAP‐*Lf*ILEP2, or the empty vector pNZ8148 (designated as Ll/ILEP^+^, Ll/AAP‐ILEP^+^, and Ll/ev, respectively) were cultured at 30 °C in 5 mL of GM17 medium (M17 broth^[31]^ with 0.5 % glucose) supplemented with 10 μg/mL chloramphenicol. When the Od_660_ reached approximately 0.6, 1 ng/mL of nisin was added to induce target protein production. At an Od_660_ of approximately 1.0, cells were harvested by centrifugation at 6,000×g at 0 °C. The cell pellets were washed twice with 5 mL of 150 mM NaCl, resuspended in 5 mL of 150 mM NaCl containing 25 % (w/v) glycerol, and stored at −80 °C until further use. Aliquots (2 μL) of the bacterial suspensions were added to 200 μL/well of semi‐defined medium (SDM, Table 3) containing either l‐BCAA or d‐BCAA in a 96‐well microplate. SDM was prepared based on the chemically defined media as described previously[^32^, ^33^] with some modifications. The concentrations of l‐BCAA added were 0.325 g/L for l–Val, 0.475 g/L for l–Leu, and 0.21 g/L for l–Ile, while those of d‐BCAA were 0.325 g/L for d‐Val, 0.475 g/L for d‐leu, and 0.21 g/L for d‐*allo*‐Ile. The cultures were incubated at 30 °C, and growth was monitored at Od_660_ using a microplate reader.

**Table 3:** Composition of the semi‐defined medium (SDM).

Glucose	10 g/L		l‐Asp	0.42 g/L
K_2_HPO_4_	3 g/L		l‐Asn^⋅^H_2_O	0.35 g/L
KH_2_PO_4_	3 g/L		l‐Glu	0.5 g/L
(NH_4_)_2_HC_6_H_5_O_7_	0.6 g/L		l‐Gln	0.39 g/L
Cysteine	50 mg/L		l‐Ser	0.34 g/L
Tween 80	1 g/L		l‐Thr	0.225 g/L
Yeast extract	5 mg/L		l‐Arg^⋅^HCl	0.125 g/L
Uracil	5 mg/L		l–Lys^⋅^HCl	0.440 g/L
Adenine	5 mg/L		l‐His	0.15 g/L
Guanine	5 mg/L		l‐Tyr	0.0181 g/L
Xanthine	5 mg/L		l‐Phe	0.275 g/L
MgCl_2_	200 mg/L		l‐Trp	0.05 g/L
CaCl_2_	50 mg/L		l‐Pro	0.675 g/L
FeCl_3_^⋅^6H_2_O	5 mg/L		l–Met	0.125 g/L
ZnSO_4_^⋅^7H_2_O	5 mg/L		l‐Ala	0.24 g/L
MnSO_4_^⋅^4H_2_O	5 mg/L		Gly	0.175 g/L
CoCl_2_^⋅^6H_2_O	2.5 mg/L			
CuSO_4_^⋅^5H_2_O	2.5 mg/L			
VOSO_4_^⋅^nH_2_O	2.5 mg/L			
Na_2_MoO_4_^⋅^nH_2_O	2.5 mg/L			

### d‐BCAA Export Assay Using *E. coli*

BW/ev2, BW/AAP2^+^. BW/ILEP2^+^, or BW/AAP‐ILEP^+^ strains were precultured in LB medium supplemented with glucose (0.2 %), ampicillin (100 μg/ml), and chloramphenicol (30 μg/ml) at 37 °C overnight. The 500 μL of culture was harvested by centrifugation (12,000 rpm, 1 min), washed twice with M9 medium, and resuspended in a 500 μL of M9‐glucose medium. The cell suspension (500 μL) was added to a 50 mL of M9 medium containing 1 mM l‐BCAAs (l–Val, l–Ile, and l–Leu), 0.02 % l‐arabinose, and antibiotics, and incubated at 37 °C with shaking. Cells and media were collected at log‐phase (~Od_600_=0.4) and stationary phase (Od_600_=1). Samples were deproteinized with trichloro acetic acid. Amino acid concentrations were determined by HPLC as described previously.[^34^, ^35^]

### Amino Acid Uptake Assay Using *E. coli*

BW/ev and BW/AAP^+^ strains were precultured in LB medium supplemented with 0.2 % glucose and 30 μg/ml chloramphenicol at 37 °C overnight. The 500 μL of culture was harvested, washed twice with M9 medium, and resuspended in 500 μL of M9 medium. Subsequently, 125 μL of the suspension was added to 6 ml of M9‐glucose medium containing 0.02 % l‐arabinose, chloramphenicol, and incubated at 37 °C with shaking. At log‐phase (Od_600_=0.6), the culture was divided into two tubes and supplemented with either water or 0.25 mM amino acid. When required, either dimethyl sulfoxide (DMSO) (Wako‐Fujifilm), 50 μM of carbonyl cyanide *m*‐chlorophenyl hydrazone (CCCP) (Wako‐Fujifilm), or 500 μM of *N, N*‐dicyclohexylcarbodiimide (DCCD) (Tokyo Kasei) was added to the medium prior to amino acid supplementation. After 10 min incubation, cells were place on ice and collected by centrifugation. Amino acids were extracted using trichloro acetic acid, and their concentrations were determined by HPLC as described previously.^[34]^

### Amino Acid Uptake Assay Using *L. Lactis*

*lactococcus lactis* strains harboring p*Lf*AAP2 (Ll/AAP^+^) or an empty vector (Ll/ev) were cultured at 30 °C in 150 mL of GM17 medium supplemented with 10 μg/mL chloramphenicol. At an Od_660_ value of approximately 0.4, 10 ng/μL nisin was added to induce *Lf*AAP expression. When Od_660_ reached ~1.0, cells were collected, washed twice with 20 mL of ice‐cold SDM without amino acids, and resuspended in 15 mL of SDM without amino acids. The suspensions were kept on ice until use. The 1 mL of the bacterial suspension was transferred to a 50 mL centrifuge tube, and 10 μL of a 50 % glucose solution was added. After incubation at 30 °C for 3 minutes, 1 mL of a 1 mM amino acid solution was added, and the mixture was incubated for 10 min at 30 °C. The reaction was terminated by adding 18 mL of ice‐cold 0.1 M liCl. After centrifugation, cell pellets were resuspended in 150 mM NaCl to achieve a cell concentration of approximately 70 mg/mL, and disrupted using a multi‐bead shocker (Yasui Kikai). The amino acid concentrations were determined using UHPLC as previously described.^[14]^ Amino acid concentrations (nmol) were normalized to the protein concentration (mg protein) in the cell extracts. Protein concentrations were measured using the method of Bradford;^[39]^ bovine serum albumin was used as the standard.

## Conflict of Interests

The authors declare no conflict of interest.

1

## Data Availability

The data that support the findings of this study are available from the corresponding author upon reasonable request.

## References

[1] W. Vollmer , D. Blanot , M. A. De Pedro , FEMS Microbiol. Rev. 2008, 32, 149–167. 18194336 10.1111/j.1574-6976.2007.00094.x

[2] F. Cava , H. Lam , M. A. De Pedro , M. K. Waldor , Cell. Mol. Life Sci. 2011, 68, 817–831. 21161322 10.1007/s00018-010-0571-8 PMC3037491

[3] P. Veiga , S. Piquet , A. Maisons , S. Furlan , P. Courtin , M. Chapot-Chartier , S. Kulakauskas , Mol. Microbiol. 2006, 62, 1713–1724. 17083466 10.1111/j.1365-2958.2006.05474.x

[4] S. Bellais , M. Arthur , L. Dubost , J.-E. Hugonnet , L. Gutmann , J. Van Heijenoort , R. Legrand , J.-P. Brouard , L. Rice , J.-L. Mainardi , J. Biol. Chem. 2006, 281, 11586–11594. 16510449 10.1074/jbc.M600114200

[5] P. Grohs , L. Gutmann , R. Legrand , B. Schoot , J. L. Mainardi , J. Bacteriol. 2000, 182, 6228–6232. 11029446 10.1128/jb.182.21.6228-6232.2000 PMC94760

[6] B. L. M. De Jonge , D. Gage , N. Xu , Antimicrob. Agents Chemother. 2002, 46, 3151–3155. 12234837 10.1128/AAC.46.10.3151-3155.2002 PMC128806

[7] A. Boniface , C. Parquet , M. Arthur , D. Mengin-Lecreulx , D. Blanot , J. Biol. Chem. 2009, 284, 21856–21862. 19542229 10.1074/jbc.M109.034363 PMC2755910

[8] H. Lam , D.-C. Oh , F. Cava , C. N. Takacs , J. Clardy , M. A. De Pedro , M. K. Waldor , Science 2009, 325, 1552–1555. 19762646 10.1126/science.1178123 PMC2759711

[9] B. L. M. De Jonge , D. Gage , N. Xu , Antimicrob. Agents Chemother. 2002, 46, 3151–3155. 12234837 10.1128/AAC.46.10.3151-3155.2002 PMC128806

[10] J. Sasabe , Y. Miyoshi , S. Rakoff-Nahoum , T. Zhang , M. Mita , B. M. Davis , K. Hamase , M. K. Waldor , Nat. Microbiol. 2016, 1, 16125. 27670111 10.1038/nmicrobiol.2016.125 PMC5074547

[11] J. Sasabe , M. Suzuki , Front. Microbiol. 2018, 9, 933. 29867842 10.3389/fmicb.2018.00933 PMC5954117

[12] A. Aliashkevich , L. Alvarez , F. Cava , Front. Microbiol. 2018, 9, 683. 29681896 10.3389/fmicb.2018.00683 PMC5898190

[13] Y. Mutaguchi , T. Ohmori , H. Akano , K. Doi , T. Ohshima , Springerplus 2013, 2, 691. 24422181 10.1186/2193-1801-2-691 PMC3884085

[14] Y. Mutaguchi , K. Kasuga , I. Kojima , Front. Microbiol. 2018, 9, 1540. 30057575 10.3389/fmicb.2018.01540 PMC6053490

[15] Y. Mutaguchi , T. Ohmori , T. Wakamatsu , K. Doi , T. Ohshima , J. Bacteriol. 2013, 195, 5207–5215. 24039265 10.1128/JB.00709-13 PMC3811583

[16] D. L. Jack , I. T. Paulsen , M. H. Saier , Microbiology (Reading) 2000, 146, 1797–1814. 10931886 10.1099/00221287-146-8-1797

[17] F. H. Wong , J. S. Chen , V. Reddy , J. L. Day , M. A. Shlykov , S. T. Wakabayashi , M. H. Saier Jr. , Microb. Physiol. 2012, 22, 105–113. 10.1159/000338542 22627175

[18] A. Vastermark , S. Wollwage , M. E. Houle , R. Rio , M. H. Saier , Proteins 2014, 82, 2797–2811. 25043943 10.1002/prot.24643 PMC4177346

[19] Ellen I. Closs , Alice Habermeier Petra Gräf , James M. Cunningham , Ulrich Förstermann , Biochemistry 1997, 36, 6462–6468. 9174363 10.1021/bi962829p

[20] K. E. J. Jungnickel , J. L. Parker , S. Newstead , Nat. Commun. 2018, 9, 550. 29416041 10.1038/s41467-018-03066-6 PMC5803215

[21] M. De Felice , C. Squires , M. Levinthal , J. Guardiola , A. Lamberti , M. Iaccarino , Molec. Gen. Genet. 1977, 156, 1–7. 340887 10.1007/BF00272245

[22] M. De Felice , M. Levinthal , M. Iaccarino , J. Guardiola , Microbiol. Rev. 1979, 43, 42–58. 379577 10.1128/mr.43.1.42-58.1979 PMC281461

[23] J. J. Anderson , D. L. Oxender , J. Bacteriol. 1977, 130, 384–392. 323236 10.1128/jb.130.1.384-392.1977 PMC235216

[24] E. A. Kutukova , V. A. Livshits , I. P. Altman , L. R. Ptitsyn , M. H. Zyiatdinov , I. L. Tokmakova , N. P. Zakataeva , FEBS Lett. 2005, 579, 4629–4634. 16098526 10.1016/j.febslet.2005.07.031

[25] Q. Liu , Y. Liang , Y. Zhang , X. Shang , S. Liu , J. Wen , T. Wen , Appl. Environ. Microbiol. 2015, 81, 7753–7766. 26319875 10.1128/AEM.02242-15 PMC4616930

[26] Y.-H. Su , W. B. Frommer , U. Ludewig , Plant Physiol. 2004, 136, 3104–3113. 15377779 10.1104/pp.104.045278 PMC523371

[27] S. Ramos , S. Schuldiner , H. R. Kaback , Proc. Natl. Acad. Sci. USA 1976, 73, 1892–1896. 6961 10.1073/pnas.73.6.1892 PMC430413

[28] J. Hermolin , R. H. Fillingame , J. Biol. Chem. 1989, 264, 3896–3903. 2521856

[29] C. Fu , W. P. Donovan , O. Shikapwashya-Hasser , X. Ye , R. H. Cole , PLoS One 2014, 9, e115318. 25551825 10.1371/journal.pone.0115318 PMC4281135

[30] T. Ito , R. Hori , H. Hemmi , D. M. Downs , T. Yoshimura , Mol. Microbiol. 2020, 113, 270–284. 31677193 10.1111/mmi.14415 PMC7060791

[31] B. E. Terzaghi , W. E. Sandine , Appl. Microbiol. 1975, 29, 807–813. 16350018 10.1128/am.29.6.807-813.1975 PMC187084

[32] B. Poolman , W. N. Konings , J. Bacteriol. 1988, 170, 700–707. 3123462 10.1128/jb.170.2.700-707.1988 PMC210711

[33] M. Rogosa , J. G. Franklin , K. D. Perry , J. Gen. Microbiol. 1961, 25, 473–82. 13742799 10.1099/00221287-25-3-473

[34] T. Ito , M. Hayashida , S. Kobayashi , N. Muto , A. Hayashi , T. Yoshimura , H. Mori , J. Biochem. 2016, 160, 345–353. 27387750 10.1093/jb/mvw043

[35] T. Ito , N. Muto , H. Sakagami , M. Tanaka , H. Hemmi , T. Yoshimura , FEBS J. 2023, 290, 2895–2908. 36695650 10.1111/febs.16734

[36] T. Ito , K. Yamamoto , R. Hori , A. Yamauchi , D. M. Downs , H. Hemmi , T. Yoshimura , Appl. Environ. Microbiol. 2019, 85, e00430–19. 30902856 10.1128/AEM.00430-19 PMC6532037

[37] S. Kumar , G. Stecher , M. Li , C. Knyaz , K. Tamura , Mol. Biol. Evol. 2018, 35, 1547–1549. 29722887 10.1093/molbev/msy096 PMC5967553

[38] J. Eberhardt , D. Santos-Martins , A. F. Tillack , S. Forli , J. Chem. Inf. Model. 2021, 61, 3891–3898. 34278794 10.1021/acs.jcim.1c00203 PMC10683950

[39] M. M. Bradford , Anal. Biochem. 1976, 72, 248–254. 942051 10.1016/0003-2697(76)90527-3

